# Efficacy of single-dose intravitreal dexamethasone implantation for retinal vein occlusion patients with refractory macular edema: A systematic review and meta-analysis

**DOI:** 10.3389/fphar.2022.951666

**Published:** 2022-09-28

**Authors:** Qiongzhen Yuan, Yunxia Gao, Yilin Liu, Hanyue Xu, Tong Wang, Ming Zhang

**Affiliations:** Department of Ophthalmology, West China Hospital, Sichuan University, Chengdu, China

**Keywords:** retinal vein occlusion, macular edema, dexamethasone implant, anti-vascular endothelial growth factor, switching treatment, meta-analysis

## Abstract

**Purpose:** To assess the functional and anatomical consequences of single-dose dexamethasone (DEX) implants for the treatment of refractory macular edema (ME) secondary to retinal vein occlusion (RVO) after anti-vascular endothelial growth factor agents.

**Methods:** A literature search of studies on switching therapy to DEX implants from anti-VEGF agents in refractory RVO patients was performed with five electronic databases (PubMed, Embase, Web of Science, MEDLINE, and Cochrane Library) prior to January 2022. The main outcomes included best-corrected visual acuity (BCVA) and central macular thickness (CMT) changes at different follow-up endpoints from baseline. All analyses were performed using Stata version 15.0.

**Results:** The final analysis included four eligible studies with a total of 99 patients. After single-dose DEX implant application, BCVA improved significantly at 2, 3, and 6 months with an average gain of −0.23 logarithm of the minimum angle of resolution (logMAR) (*p* = 0.004), −0.20 logMAR (*p* = 0.027), and -0.09 logMAR (*p* = 0.021), respectively. Mean CMT reduction was also significant from baseline to 2 months (-241.89 μm, *p* < 0.001), 3 months (−222.61 μm, *p* < 0.001), and 6 months (−90.49 μm, *p* < 0.001). No serious adverse events were observed in any of the included studies.

**Conclusion:** This meta-analysis showed that RVO patients with refractory ME could benefit significantly from switching therapy to DEX implantation, with efficacy lasting 6 months after a single-dose application. Intravitreal DEX implantation is a safe and effective option for refractory cases.

## 1 Introduction

Retinal vein occlusion (RVO) caused by occlusion of the retinal venous system is the second most frequent vascular disease after diabetic retinopathy. According to the occlusion location, it is classified as central retinal vein occlusion (CRVO), hemiretinal vein occlusion (HRVO), or branch retinal vein occlusion (BRVO) (Laouri et al., 2011; Scott et al., 2012; Glanville et al., 2014). Macular edema (ME) is the primary cause of bad vision in RVO patients, presenting in 5%–15% of eyes over a period of 1 year in RVO (Campochiaro et al., 2008; Lee et al., 2017).

Initially, treatment alternatives for ME consisted of observation, intravitreal triamcinolone acetonide, laser photocoagulation, and vitrectomy. The development of drugs, especially intravitreal anti-vascular endothelial growth factor (VEGF) drugs, has opened new avenues for the treatment of RVO. More recently, intravitreal anti-VEGF injections have been the first-line therapy for ME secondary to RVO, which require a loading phase of three consecutive monthly injections (Berger et al., 2015; Moisseiev and Loewenstein, 2020). Most clinical trials have demonstrated that intravitreal anti-VEGF therapy is effective in improving vision and reducing ME with relatively few complications (Brown et al., 2010; Campochiaro et al., 2011; Varma et al., 2012; Campochiaro et al., 2014; Clark et al., 2016; Bajor et al., 2017). However, this is not always the case; a subset of patients may not respond to anti-VEGF therapy immediately or may develop rebound ME despite extensive monthly injection (Matsumoto et al., 2007; Yasuda et al., 2011).

In fact, the pathogenesis of ME is extremely complicated, and factors other than VEGFs should not be overlooked. Some studies have shown that various cytokines, including interleukin-8 and interleukin-6, play significant roles in ME as well (Kang et al., 2001; Noma et al., 2009; Fonollosa et al., 2010). Intravitreal corticosteroids are thought to be effective agents for ME as they can inhibit the expression of VEGFs, downregulate inflammatory stimuli, inhibit leukocyte migration, and enhance the function of the blood–retinal barrier (Kern, 2007; Zhang et al., 2008). Dexamethasone (DEX) has the highest clinical effectiveness of any corticosteroid administrated in ophthalmological practice. The sustained-release 0.7 mg DEX implant (Ozurdex; Allergan, Irvine, CA, United States) is a biodegradable device developed to deliver DEX over a period of 6 months. This implant has been approved by the United States Food and Drug Administration for diabetic ME therapy, posterior noninfectious uveitis, and macular edema in RVO. Its safety and effectiveness in treating of naïve RVO-induced ME have been proven, with efficacy lasting for 6 months after a single-dose therapy (Haller et al., 2010; Haller et al., 2011).

Switching therapy to intravitreal DEX implants is recommended for patients with refractory ME secondary to diabetic retinopathy and RVO. The safety and efficacy of switching therapy from intravitreal anti-VEGF drugs to DEX implants in diabetic ME patients have been well proven (Shah et al., 2016; Castro-Navarro et al., 2019; Yuan et al., 2022). Although several studies have estimated the efficacy and safety of intravitreal DEX implantation for the treatment of patients with refractory ME, consistent conclusions have not been reached, and a comprehensive synthesis of existing data has not been published (Ozkok et al., 2015; Chiquet et al., 2016; Wallsh et al., 2016; Hanhart and Rozenman, 2017; Manousaridis et al., 2017; Georgalas et al., 2019). Thus, we conducted this meta-analysis to systematically investigate the retinal anatomical and visual outcomes of refractory RVO patients following switching therapy to intravitreal DEX implants from anti-VEGF drugs.

## 2 Methods

### 2.1 Literature search

We thoroughly searched five electronic databases (PubMed, Embase, Web of Science, MEDLINE, and Cochrane Library) prior to January 2022 and performed the literature search through a combination of medical subject headings with keywords of the following terms: “dexamethasone or Ozurdex,” “retinal vein occlusion or RVO,” “resistant,” “switching,” “non-response,” “refractory,” “recalcitrant,” “recurrent,” “conversion,” and “persistent.” Studies in English on switching therapy to DEX implants from anti-VEGF agents in refractory RVO patients were reviewed. The reference lists from the identified articles and additional literature were further investigated to identify any relevant studies. This meta-analysis was performed following the Preferred Reporting Items for Systematic Reviews and Meta-Analyses (PRISMA) Checklist (Moher et al., 2009).

### 2.2 Eligibility criteria

Inclusion criteria: 1) RVO patients older than 18 years; 2) refractory ME after anti-VEGF treatment; 3) with 6 months of follow-up after receiving single-dose DEX implant; 4) discontinued anti-VEGF treatment during DEX implant (Ozurdex) therapy; 5) both primary measures (best-corrected visual acuity [BCVA] and central macular thickness [CMT]) were demonstrated as mean ± standard deviation; and 6) written informed consent was obtained in advance from each participant, and studies were conducted based on principles of the Declaration of Helsinki. Studies were excluded if patients had other causes of ME, such as age-related macular disease, diabetic retinopathy, or noninfectious posterior uveitis. Reviews, case reports with fewer than five patients, letters without data, and conference abstracts were also excluded. We included the most recent result when the same research data were demonstrated in various publications.

### 2.3 Data extraction and quality assessment

Two investigators (QY and YG) independently assessed the available articles and extracted data from each study using a pre-established extraction table, including publication information (first author, publication time, country, RVO subtype, and study type), patient characteristics (sample size and average age), therapy information (type of anti-VEGF drug prior to conversion, number of injections, and follow-up duration), and efficacy parameters (BCVA and CMT). The Downs and Black checklist (Downs and Black, 1998) was adopted to independently assess the methodological quality of all selected studies by the same investigators. The checklist has an overall score range of 0–28 and is considered suitable for evaluating both randomized and non-randomized trials. The higher the score, the better the methodological quality. The scoring range reflects the corresponding quality level: poor (0–14), fair (15–19), good (20–25), and excellent (26–28) (Hooper et al., 2008). All enrolled studies were graded as having fair quality (score range: 16–19). We resolved any disagreements through discussion and consultation.

### 2.4 Outcome measures

The primary measures were mean BCVA and CMT changes at various follow-up endpoints from baseline following the therapy switch. We transposed BCVA data to the logarithm of the minimum angle of resolution (logMAR) when demonstrated in the Early Treatment Diabetic Retinopathy Study (ETDRS) letter score or Snellen fraction. The safety evaluation included ocular and systemic adverse events (AEs) during intravitreal DEX implant application.

### 2.5 Statistical analyses

Data analyses were conducted using Stata software (version 15.0; Stata Corporation, College Station, TX, United States). The effect size of continuous data is presented as the mean difference and 95% confidence intervals (CIs). We used the Cochran Q test and the statistical value I^2^ to estimate statistical heterogeneity. When *p* > 0.1 and I^2^<50%, studies were considered to have acceptable heterogeneity. We assessed the publication bias of the included studies using Begg’s and Egger’s tests. We conducted a sensitivity analysis by adopting the leave-one-out approach. Random-effects models were applied to analyze all the data because they produce highly conservative estimates when residual heterogeneity is present. A two-sided *p* ≤ 0.05 was regarded as statistically significant in our analysis.

## 3 Results

### 3.1 Study selection and description of studies

**FIGURE 1:**
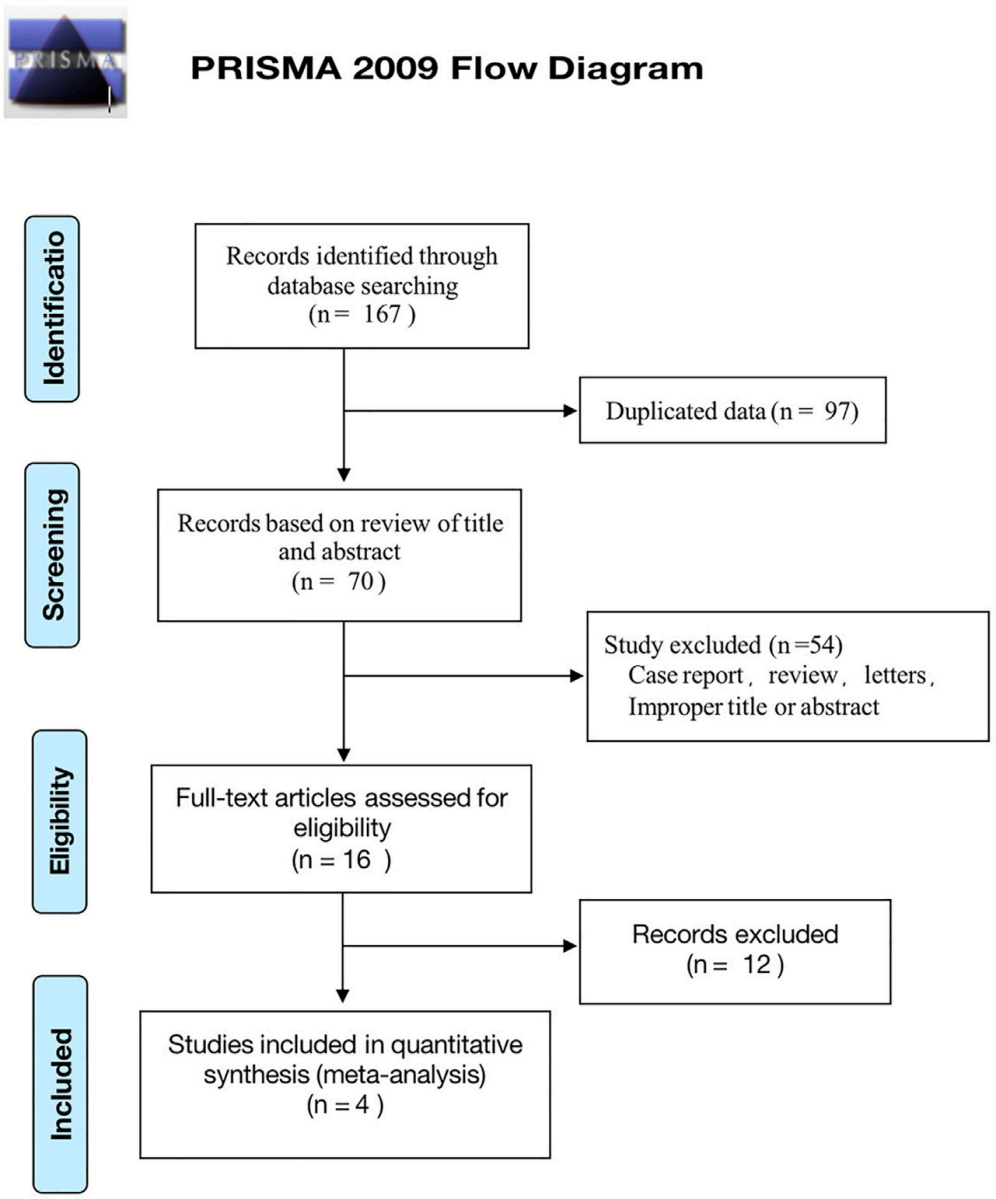
Preferred Reporting Items for Systematic Reviews and Meta-Analyses (PRISMA) flow chart of study identification and selection process.

A total of 167 studies were identified from our original strategy literature search, of which 97 were ruled out as duplications. Subsequently, 54 studies were excluded due to improper titles or abstracts, or were case reports, reviews, or letters after viewing the titles and abstracts. Among the remaining 16 articles that underwent a full review, 12 were excluded in compliance with the eligibility criteria. Finally, a total of four articles were eligible for our analysis (Alshahrani et al., 2016; Lee et al., 2017; Manousaridis et al., 2017; Georgalas et al., 2019). Among the four observational studies, one was prospective and three were retrospective in design. The PRISMA flow diagram of the study selection process is illustrated in Figure 1.

### 3.2 Baseline characteristics

The main characteristics of the eligible studies are summarized in Table 1 and Supplementary Table S1. A total of 99 patients (99 eyes) were included in our analyses, with sample sizes ranging from 11 to 38 patients and a mean age ranging from 65.92 to 68.04 years. ME was secondary to BRVO in 72 eyes, CRVO in 26 eyes, and HRVO in one eye. The average baseline logMAR BCVA ranged from 0.53 to 0.88, and CMT ranged from 504.00 to 572.22 μm. The mean number of injections of intravitreal anti-VEGF drugs was 3.83–9 times prior to switching therapy. All participants underwent one intravitreal DEX implantation and were followed up for 6 months.

**TABLE 1:** General characteristics of four studies included in this meta-analysis.

Author	Year	Location	Study design	Subtype RVO	Eyes [patients]	Age (y); mean ± SD [range]	Anti-VEGF type before switching	Number of injections before switching	Downs and Black score
Lee et al. (2017)	2017	Korean	Retrospective (self-controlled)	BRVO	38 [38]	67.76 ± 10.27	IVB	6.32 ± 4.66	17
Manousaridis et al. 2017	2017	Austria	Retrospective (self-controlled)	CRVO/BRVO/HRVO	11 [11]	N/A	IVR	9 [6–16]	16
Georgalas et al. 2019	2019	Greece	Retrospective (self-controlled)	CRVO/BRVO	23 [23]	65.92 ± 9.99	IVR or IVA	5.22 ± 2.78	19
Alshahrani et al. 2016	2016	Arabia	Retrospective (self-controlled)	CRVO/BRVO	27 [27]	68.04 ± 10.22	N/A	3.83	16

CRVO, central retinal vein occlusion; HRVO, hemiretinal vein occlusion; BRVO, branch retinal vein occlusion; IVB, intravitreal bevacizumab; IVR, intravitreal ranibizumab; IVA, intravitreal aflibercept; N/A, not available; SD, standard deviation; VEGF, vascular endothelial growth factor; DEX, dexamethasone.

### 3.3 Best-corrected visual acuity

**FIGURE 2:**
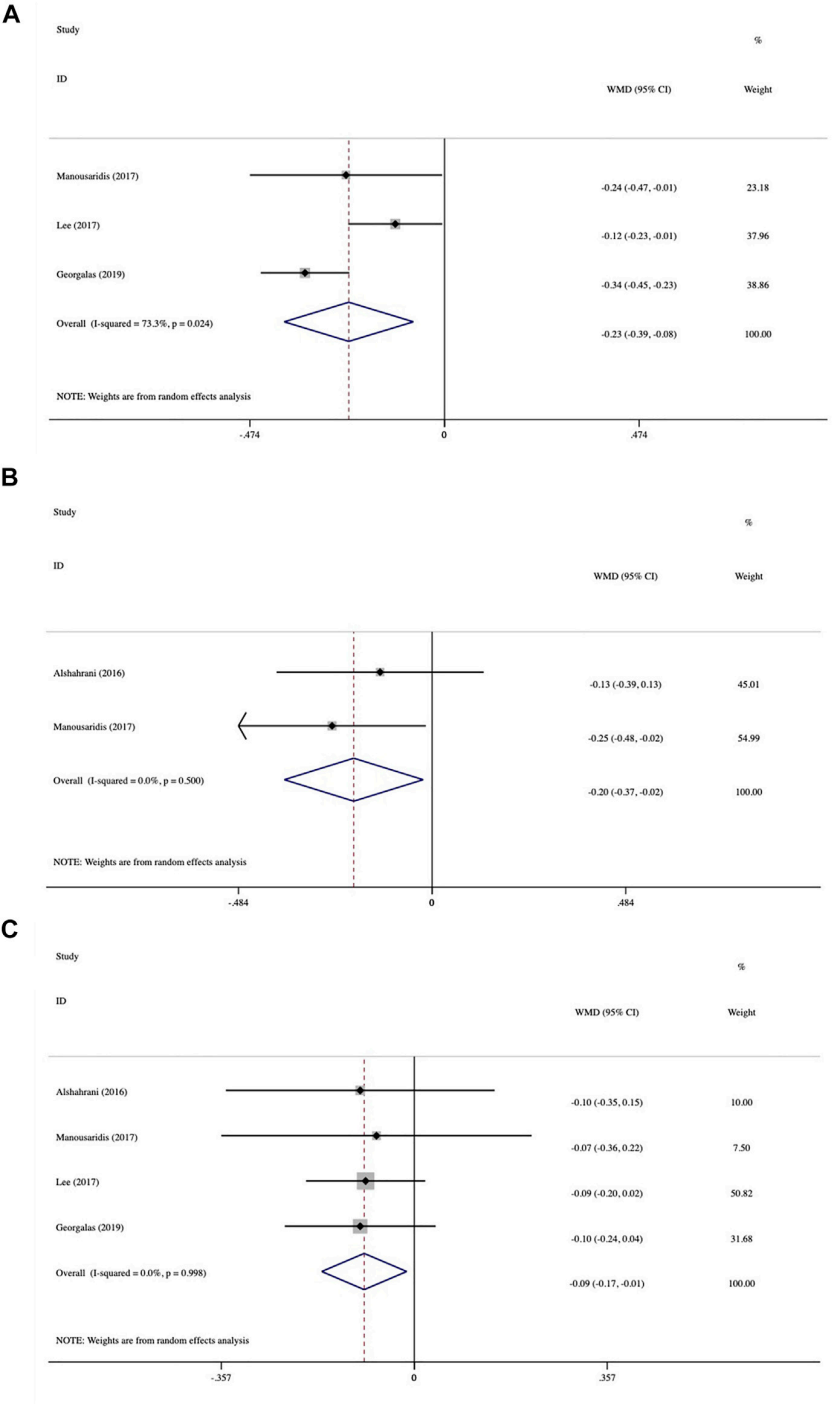
Forest plots of mean best-corrected visual acuity (BCVA) changes at various follow-up endpoints after switching therapy **(A)** at 2 months, **(B)** at 3 months, and **(C)** at 6 months.

The investigation of mean BCVA change between baseline and different follow-up endpoints was demonstrated in the forest plots (Figure 2). We included three studies that evaluated the mean BCVA change at month 2. The pooled results demonstrated a significant change in BCVA, with an average gain of −0.23 logMAR (95% CI: −0.39 to −0.08, *p* = 0.004; Figure 2A). The evaluation of BCVA change at 3 months was conducted in two studies and showed significant improvement with an average of −0.20 logMAR (95% CI: −0.37 to −0.02, *p* = 0.027; Figure 2B). In four studies, at 6 months, the average BCVA significantly improved by −0.09 logMAR (95% CI: −0.17 to −0.01, *p* = 0.021; Figure 2C). The greatest mean BCVA improvement occurred at 2 months, and significant BCVA improvement persisted until month 6. No inter-study heterogeneity was found in studies at 3 months (I^2^ = 0%, *p* = 0.5) or 6 months (I^2^ = 0%, *p* = 0.998).

### 3.4 Central macular thickness

**FIGURE 3:**
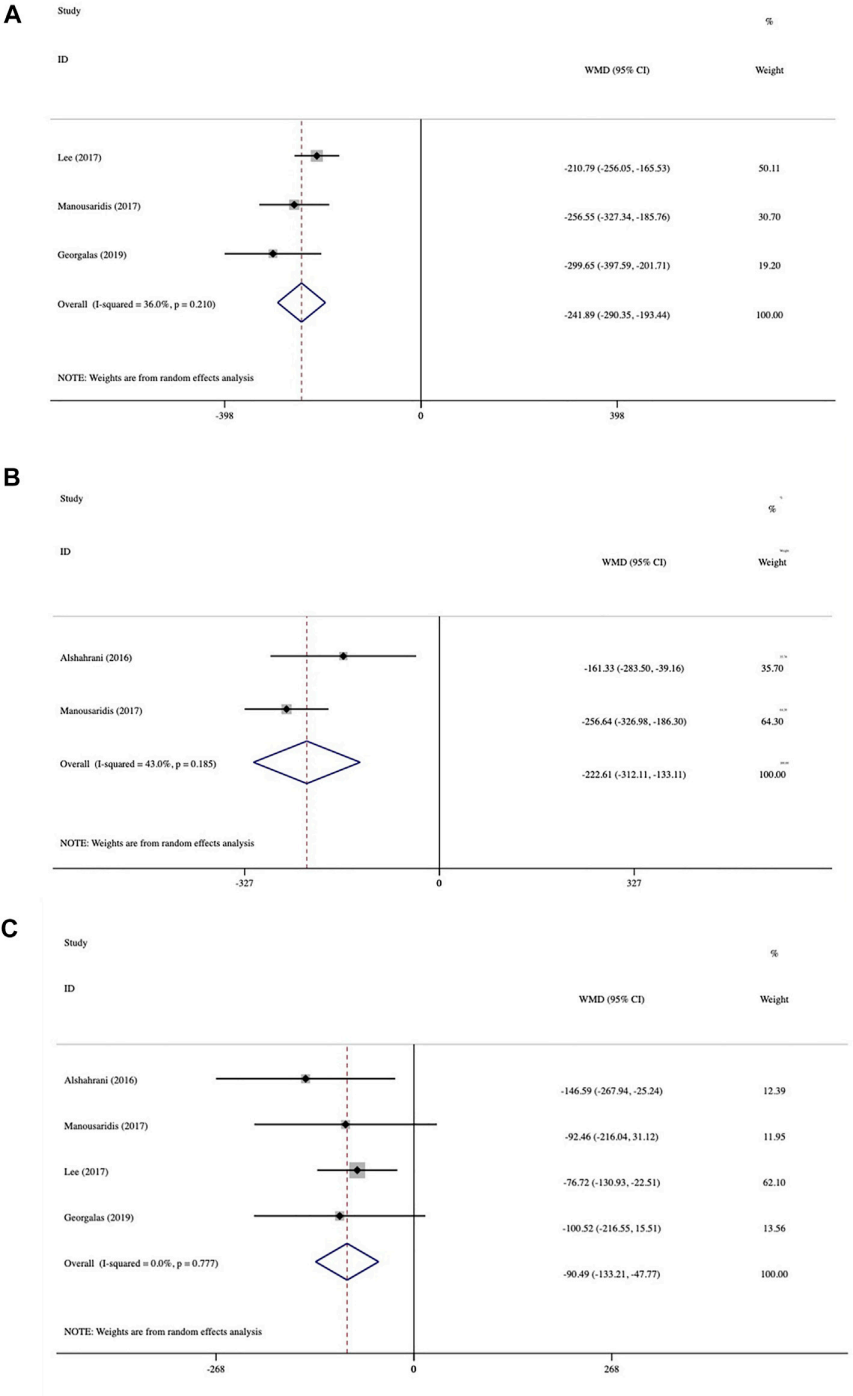
Forest plots of mean central macular thickness (CMT) changes at various follow-up endpoints after switching therapy **(A)** at 2 months, **(B)** at 3 months, and **(C)** at 6 months.

The average CMT changes at various follow-up times from baseline are presented in Figure 3. In three studies at 2 months, CMT was reduced dramatically from baseline by 241.89 μm (95% CI: −290.35 to −193.44, *p* < 0.001; Figure 3A). Data analysis from two trials at 3 months showed an average change of 222.61 μm (95% CI: −312.11 to −133.11, *p* < 0.001; Figure 3B). Mean CMT change was analyzed in four studies at 6 months, which demonstrated an average reduction of 90.49 μm (95% CI: −133.21 to −47.77, *p* < 0.001; Figure 3C). The greatest mean CMT reduction occurred at 2 months, and the significant reduction lasted until 6 months. No statistically significant inter-study heterogeneity was observed among studies at 2 months (I^2^ = 36%, *p* = 0.21), 3 months (I^2^ = 43%, *p* = 0.185), or 6 months (I^2^ = 0.0%, *p* = 0.777).

### 3.5 Subgroup analysis

Subgroup analysis by occlusion position (BRVO in 72 eyes and CRVO in 26 eyes) included only three follow-up time points because of the limited number of studies (Table 2). The assessment of BCVA change in the two subgroups is shown in Supplementary Figure S1. Patients with BRVO achieved significant BCVA improvement at all three follow-up time points, with an average of −0.21 logMAR at 2 months (95% CI: −0.31 to −0.12, *p* < 0.001), −0.22 logMAR at 3 months (95% CI: −0.34 to −0.11, *p* < 0.001), and −0.09 logMAR at 6 months (95% CI: −0.16 to −0.02, *p* = 0.013). In the CRVO subgroup, the average BCVA improvement was only significant at 2 months (−0.40 logMAR; 95% CI: −0.57 to -0.23, *p* < 0.001), while it was not statistically significant at 3 months (-0.15 logMAR; 95% CI: −0.47 to 0.17, *p* = 0.359) and 6 months (−0.10 logMAR; 95% CI: −0.29 to 0.08, *p* = 0.280).

**TABLE 2:** Subgroup analysis of BCVA (logMAR) and CMT (μm) outcomes (mean and 95% confidence intervals).

	BRVO	CRVO
BCVA at 2 months	−0.21 (−0.31 to −0.12)	−0.40 (−0.57 to −0.23)
BCVA at 3 months	−0.22 (−0.34 to 0.11)	−0.15 (−0.47 to 0.17)
BCVA at 6 months	−0.09 (−0.16 to −0.02)	−0.10 (−0.29 to 0.08)
CMT at 2 months	−211.92 (−245.72 to −178.13)	−387.65 (−506.45 to −268.85)
CMT at 3 months	−192.07 (−246.16 to −137.99)	−283.73 (−436.93 to −150.52)
CMT at 6 months	−85.72 (−127.21 to −44.23)	−122.55 (−252.42 to 7.32)

BCVA, best-corrected visual acuity; CMT, central macular thickness; BRVO, branch retinal vein occlusion; CRVO, central retinal vein occlusion; logMAR, logarithm of the minimum angle of resolution.

The mean CMT decrease at all three follow-up endpoints was statistically significant in the BRVO subgroup, whereas it was only significant at 2 and 3 months in the CRVO subgroup. The CRVO subgroup showed a greater mean decrease than the BRVO subgroup (Supplementary Figure S2). In the CRVO subgroup, mean CMT reduced significantly by 387.65 μm (95% CI: −506.45 to −268.85, *p* < 0.001) at month 2, 283.73 μm (95% CI: −436.93 to −130.52, *p* < 0.001) at month 3, and 122.55 μm (95% CI: −252.42 to 7.32, *p* < 0.001) at month 6. Mean reduction in the BRVO subgroup was 211.92 μm (95% CI, −245.72 to −178.13, *p* < 0.001) at 2 months, 192.07 μm (95% CI, −246.16 to −137.99, *p* < 0.001) at month 3, and 85.72 μm (95% CI, −127.21 to −44.23, *p* = 0.064) at month 6. The greatest average CMT reduction in both the groups occurred at 2 months.

### 3.6 Publication bias

No evidence of possible publication bias was demonstrated when assessed using Begg’s test (BCVA, *p* = 0.734; CMT, *p* = 0.712) and Egger’s test (BCVA, *p* = 0.734; CMT, *p* = 0.225). Sensitivity analysis manifested that no single trial had a significant effect on the pooled results, demonstrating that the results were stable.

### 3.7 Safety

No serious ocular or systematic AEs were reported in any included studies. Among the AEs observed, elevated intraocular pressure (IOP) and cataract progression were the most frequent. The most significantly increased IOP could be controlled to normal with topical antiglaucoma medication. Only one patient underwent IOP-lowering surgery in two studies (cyclodestructive procedure and trabeculectomy, respectively) (Manousaridis et al., 2017; Teja et al., 2019).

## 4 Discussion

This study, to the best of our knowledge, is the first to comprehensively assess the efficacy of single-dose intravitreal DEX implantation for the treatment of RVO in patients with persistent refractory ME to anti-VEGF agents. Although RVO is the second most frequent retinal vascular disease, only a limited number of publications have reported switching therapy from anti-VEGF to DEX implants for recalcitrant RVO. Four trials involving 99 patients (99 eyes) were included in our meta-analysis. Six months of follow-up provided an adequate time window to evaluate the effectiveness of single-dose DEX. Most eligible studies showed significant BCVA gains and CMT reductions, which is in line with our pooled analysis. This meta-analysis demonstrated that refractory RVO participants could benefit greatly by switching to the DEX implant, with significant BCVA and CMT improvement at 2, 3, and 6 months after one intravitreal injection of the DEX implant. Additionally, both BCVA and CMT showed the greatest improvement at 2 months. As reported, the DEX implant had its peak concentration at 2 months and maximum efficacy between the first and third months (Chang-Lin et al., 2011; Pacella et al., 2013).

Although high levels of VEGF have been reported as a major factor in the development of ME secondary to vascular retinopathies, including RVO, other factors, such as inflammation, should not be ignored. For refractory RVO patients, inflammatory factors other than VEGF may play a much more crucial role in the pathogenesis of ME. The efficacy of DEX implantation in refractory cases might be attributed to its pharmacological mechanism. Corticosteroids can inhibit the production of VEGF, prostaglandin, and some pro-inflammatory cytokines. Corticosteroids can also reduce vascular permeability and leukocyte migration as well as stabilize vascular endothelial cell tight junctions (Chang-Lin et al., 2011; Rezar-Dreindl et al., 2017). DEX has the highest effect among any corticosteroids administrated in ophthalmological practice.

Subgroup analysis demonstrated that the BRVO subgroup had significant BCVA and CMT improvement at all three follow-up times, whereas the CRVO subgroup showed significant improvement in BCVA only at 2 months and significant improvement in CMT only at 2 and 3 months. Although the mean CMT reduction in the CRVO subgroup was not statistically significant at 6 months, the CMT reduction in the CRVO subgroup was greater than that in the BRVO subgroup at all three follow-up endpoints. A possible reason may be that the baseline conditions (both anatomically and functionally) of patients with CRVO were generally worse than those of patients with BRVO. CRVO eyes with poor ME and strong CMT at baseline may have greater potential for CMT improvement, but functional gain is limited due to more severe retinal damage.

Serious AEs were not reported in any of the available articles. Cataract progression and increased IOP were the most frequent AEs. Most of the increased IOP could be controlled to normal levels with topical anti-glaucoma medication alone. It has been reported that patients who undergo DEX implantation are more likely to develop cataract progression and ocular hypertension than those undergoing anti-VEGF treatments (He et al., 2018). Therefore, caution should be exercised with intravitreal DEX implantation in patients with clear lenses and/or a high IOP.

This meta-analysis had several limitations. First, the final analysis included a limited number of studies, and no randomized controlled trials were available. Second, some trials had fewer than 20 samples, which may overestimate the efficacy of conversion therapy to DEX implantation. Third, all eligible studies were of fair quality. Finally, we converted BCVA data to logMAR units from the Snellen acuity fraction and/or ETDRS letter scores in some studies for analysis.

## 5 Conclusion

In conclusion, our analysis showed positive evidence for switching therapy to intravitreal DEX implantation in eyes with refractory ME secondary to RVO, which was effective for 6 months after a single-dose application. DEX implants are an effective and safe option for treating refractory RVO.

## Data Availability

The original contributions presented in the study are included in the article/Supplementary Material; further inquiries can be directed to the corresponding author.

## Supplementary material

Supplementary Figure 1 Forest plots demonstrating mean best-corrected visual acuity (BCVA) changes in branch retinal vein occlusion (BRVO) and central retinal vein occlusion (CRVO) subgroups after switching treatment **(A)** at 2 months, **(B)** at 3 months, and **(C)** at 6 months.

Supplementary Figure 2 Forest plots demonstrating mean central macular thickness (CMT) changes in branch retinal vein occlusion (BRVO) and central retinal vein occlusion (CRVO) subgroups after switching treatment **(A)** at 2 months, **(B)** at 3 months, and **(C)** at 6 months.

## Abbreviations

AEs, adverse events; BCVA, best-corrected visual acuity; BRVO, branch retinal vein occlusion; CI, confidence interval; CMT, central macular thickness; CRVO, central retinal vein occlusion; DEX, dexamethasone; ETDRS, Early Treatment Diabetic Retinopathy Study; HRVO, hemiretinal vein occlusion; IOP, intraocular pressure; logMAR, logarithm of the minimum angle of resolution; ME, macular edema; RVO, retinal vein occlusion; VEGF, vascular endothelial growth factor.
