# Supplementary material for: Efficacy of single-dose intravitreal dexamethasone implantation for retinal vein occlusion patients with refractory macular edema: A systematic review and meta-analysis
Source: Front Pharmacol. 2022 Sep 28;13:951666. doi: 10.3389/fphar.2022.951666 (PMC9554209; doi:10.3389/fphar.2022.951666)
Supplement: Supplementary file 1 [file DataSheet1.pdf]

Supplementary

(A)

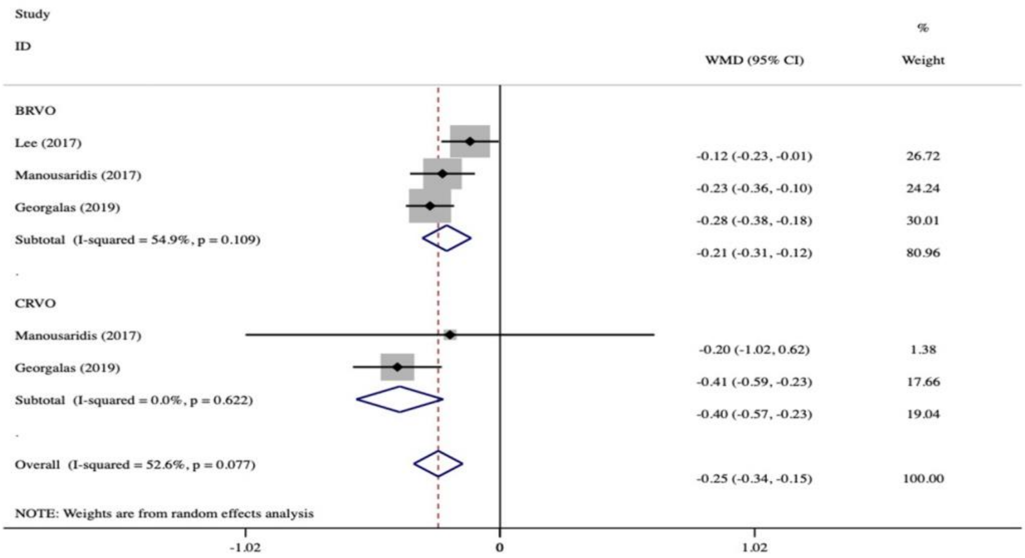

(B)

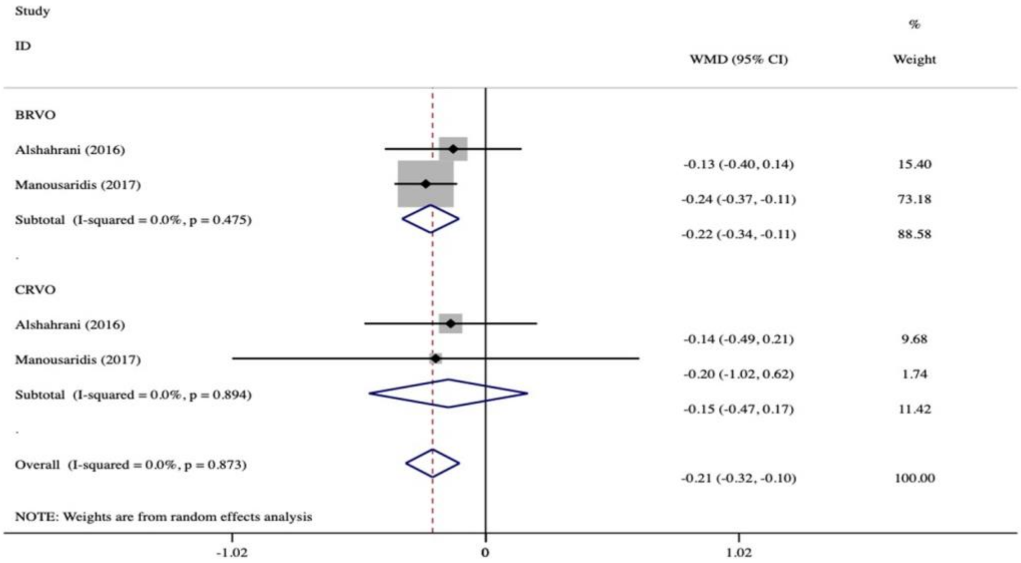

(C)

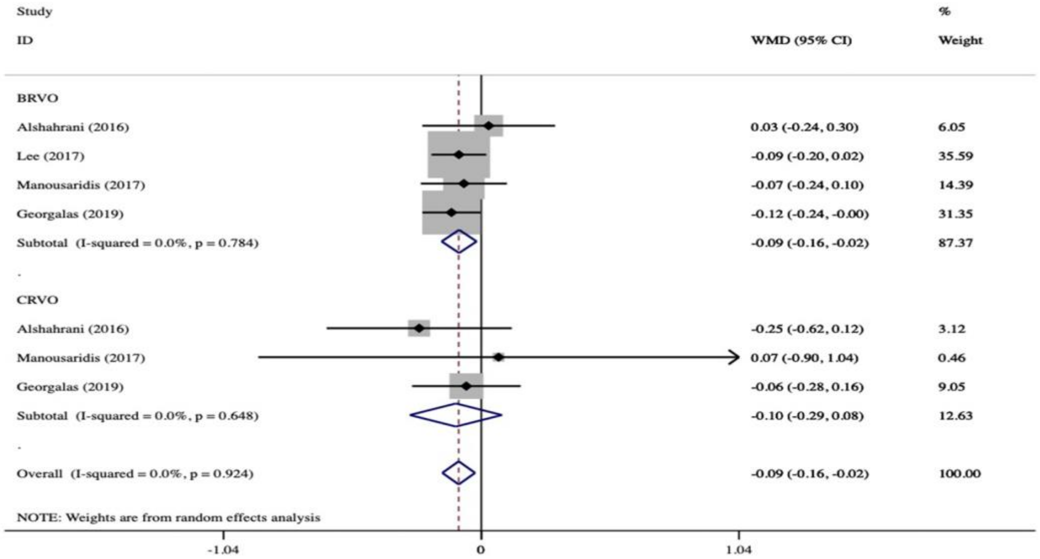

Figure S1. Forest plots demonstrating mean best-corrected visual acuity (BCVA) changes in branch retinal vein occlusion (BRVO) and central retinal vein occlusion (CRVO) subgroups after switching treatment **(A)** at 2 months, **(B)** at 3 months, and **(C)** at 6 months.

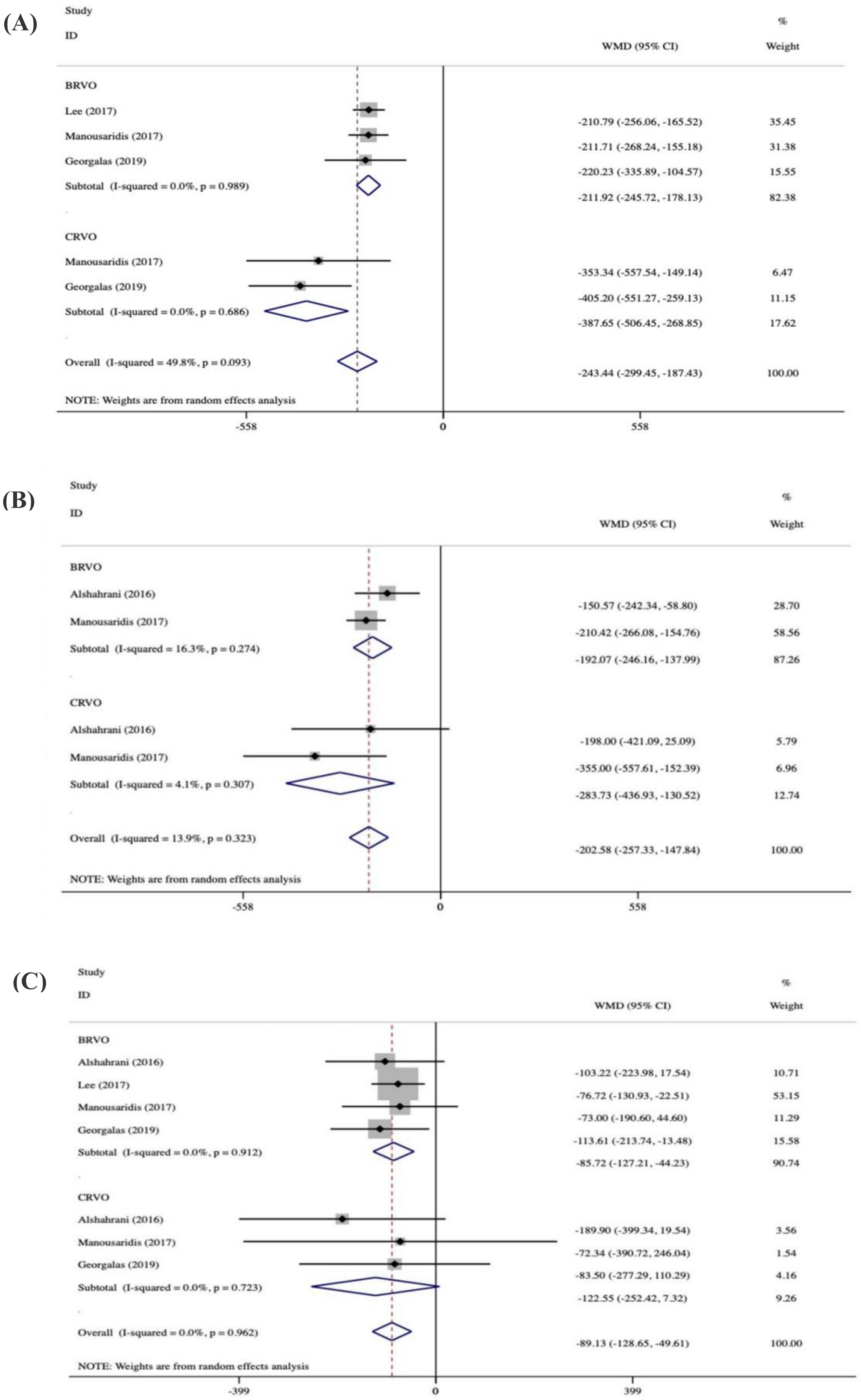

Figure S2. Forest plots demonstrating mean central macular thickness (CMT) changes in branch retinal vein occlusion (BRVO) and central retinal vein occlusion (CRVO) subgroups after switching treatment **(A)** at 2 months, **(B)** at 3 months, and **(C)** at 6 months.

Table S1. Study characteristics of the four studies in the meta-analysis.

Author	Year	Adverse	Definition of treatment resistance	Inclusion/exclusion criteria
Lee et al	2017	A notable increase in IOP (>10 mmHg from baseline) was noted in five eyes (13.1%) and controlled with topical anti-glaucoma medication. No other complications were observed. Two cases showed notable cataract progression and underwent cataract extraction 6 and 8 months after DEX implantation.	Refractory ME was defined as: (1) no improvement or worsening visual acuity, (2) CSMT reduction<150 μm, and CSMT >300 μm.	Inclusion criteria: (1) initially treated with two or more consecutive bevacizumab, (2) refractory to bevacizumab and (3) followed-up for at least 6 months after DEX implantation. Exclusion criteria: severe media opacity, previous vitreoretinal surgery, intraocular inflammation, and other disorders that may have influenced macular function (e.g., exudative age-related macular degeneration, proliferative diabetic retinopathy, and epiretinal membrane), visual acuity worse than 20 / 400.
Manousaridis et al	2017	One patient had dramatic IOP elevation, which could not be be controlled by topical and systemic IOP-lowering medication, so a cyclodestructive procedure was performed. One patient developed cataract during follow-up. No other intraoperative or postoperative complications like retinal detachment, vitreous hemorrhage, endophthalmitis, or lens injury in phakic patients were observed.	Macular edema was considered as refractory if 4 weeks after the last ranibizumab injection no change of the pattern of macular fluid was noticed on OCT and no improvement of at least two Snellen lines of the BCVA was documented, despite at least three monthly ranibizumab injections, excluding the loading phase of three consecutive monthly injections.	Inclusion criteria : (a) no other retinal pathology; (b) no cataract or other intraocular surgery performed within 6 months prior to treatment with Ozurdex;(c) nonvitrectomized eyes (d) no retinal laser photocoagulation performed within 6months prior to treatment with Ozurdex;(e) no presence of glaucoma or ocular hypertension (f) follow-up data of the BCVA, CMT and IOP available at 2, 3, and 6 months after treatment.
Georgalas et al	2019	Two of three patients with cataract progression underwent surgery during follow-up. Five patients presented with ocular hypertension (IOP < 30 mmHg) and were successfully treated with antihypertensive topical therapy.	A poor response was considered the presence of persistent ME (CRT>250 μm) after at least five injections, with subsequent lack of improvement or deterioration in BCVA. The first three injections were administered at monthly intervals, and subsequent injections in a Pro Renata (PRN) regimen (monthly for ranibizumab and bimonthly for aflibercept).	Inclusion criteria: CRVO or BRVO patients with refractory ME after at least five anti-VEGF injections, aged >18 years. Exclusion criteria: baseline visual acuity worse than 1.5 logMAR, previous intravitreal. implant, history of vitreoretinal surgery, manifest glaucoma or ocular hypertension, epiretinal membrane, retinal neovascularization, massive retinal or macular ischaemia, vitreous haemorrhage or severe lens opacity, previous laser photocoagulation treatment. Per Os or via any other route (including ocular drops) corticosteroid therapy (during the last 3 months), and known steroid responders as well as diabetic patients were also excluded.
Alshahrani et al.	2016	Increased IOP was in 14 (26%) eyes controlled with topical anti-glaucoma medication. Cataract progression was noted in 1 (1.8%) eye.	Refractory ME was defined as no improvement of 2 or more lines in Snellen BCVA and of the CMT that remained above 350 mm despite monthly injections for at least 6 months of anti-VEGF agents.	Inclusion criteria: refractory ME due to retinal vascular diseases were included, follow-up visits were performed at 1 month, 3 months, and 6 months after injection of the DEX implant. Exclusion criteria: a history of glaucoma in the study eye, patients who were not compliant. with follow-up appointments, laser treatment in the study eye within the previous 3 months, or patients who had any reason for visual acuity loss.

BCVA, best-corrected visual acuity; CMT, central macular thickness; CFT, central foveal thickness; CRT, central retinal thickness; CSMT, central subfield macular thickness; BRVO, branch retinal vein occlusion; CRVO, central retinal vein occlusion; HRVO, hemiretinal vein occlusion; VALS, visual acuity letter score; IOP, intraocular pressure; N/A, not available; OCT, optical coherence tomography; SD-OCT, spectral-domain optical coherence tomography; VA, visual acuity; VEGF, vascular endothelial growth factor.ME, macular edema; ETDRS, Early Treatment of Diabetic Retinopathy Study; DEX, dexamethasone.
